# Cyclic extranasal neurostimulation for dry eye disease: a 4-week stimulation and 6-week intermission regimen based on neural adaptation dynamics

**DOI:** 10.1186/s40662-025-00466-w

**Published:** 2025-12-02

**Authors:** Xirenayi Zhuoruo, Yike Li, Yiting Sun, Chongyang Mu, Hongjing Sun, Tao Jiang, Chunyang Wang, Ning Wang, Xiuming Jin

**Affiliations:** Eye Center of Second Affiliated Hospital, School of Medicine, Zhejiang Provincial Key Laboratory of Ophthalmology, Zhejiang Provincial Clinical Research Center for Eye Diseases, Zhejiang Provincial Engineering Institute on Eye Diseases, Zhejiang University, 1 Xihu Avenue, Hangzhou, 310009 China

**Keywords:** Cyclic regimen, Dry eye disease, Neural sensitivity, Neurostimulation, Tear secretion

## Abstract

**Background:**

Extranasal neurostimulation is a promising therapy for aqueous-deficient dry eye (ADDE), but neural adaptation and optimal treatment duration remain poorly characterized. This study investigated duration-dependent neural sensitivity dynamics to formulate an optimized cyclic treatment regimen.

**Methods:**

In this prospective, randomized, open-label trial, 50 ADDE patients (Schirmer test: 2–5 mm) were assigned to 2-week or 4-week bilateral extranasal neurostimulation (twice daily). Participants were followed for 6 weeks post-treatment. The primary outcomes were neural sensitivity changes (Δ Schirmer) and short-term recovery (6 weeks after stimulation cessation). Secondary endpoints included subjective symptoms and objective signs.

**Results:**

Distinct neural adaptation patterns were observed. The 4-week group exhibited reversible neural adaptation, with an attenuated stimulation response at treatment completion (Δ Schirmer: 1.18 ± 4.17 mm, *P* = 0.051, Cohen's d = 0.28) that fully recovered after the 6-week intermission (post-stimulation Schirmer: 10.90 ± 7.29 mm vs. initial 12.42 ± 6.67 mm, *P* = 0.90, η^2^p = 0.092). This reversible adaptation was associated with sustained improvements in tear film stability [fluorescent tear film break-up time (FBUT): 6.27 ± 2.49 s vs. baseline 3.18 ± 1.36 s, *P* < 0.001, η^2^p = 0.235] and tear volume [tear meniscus height (TMH): 0.25 ± 0.05 mm vs. 0.19 ± 0.02 mm, *P* < 0.001, η^2^p = 0.26]. Critically, this reversible adaptation pattern was exclusive to the 4-week protocol. The 2-week protocol showed preserved stimulation responses during treatment (Δ Schirmer: 3.22 ± 6.54 mm, *P* < 0.001, Cohen's d = 0.49) but developed persistent neural hyposensitivity (8.76 ± 5.95 mm vs. initial 14.88 ± 8.69 mm, *P* < 0.001, η^2^p = 0.24). Its clinical benefits, though significant for FBUT (5.20 ± 2.81 s vs. baseline 2.86 ± 1.01 s, *P* < 0.001, η^2^p = 0.109), were less pronounced than in the 4-week group, and TMH showed no sustained increase (0.21 ± 0.04 mm vs. baseline 0.19 ± 0.03 mm, *P* = 0.11, η^2^p = 0.112). Both protocols demonstrated excellent safety and high patient satisfaction.

**Conclusions:**

Based on neural adaptation and recovery characteristics, a cyclic regimen of 4-week extranasal neurostimulation followed by a 6-week intermission is proposed as a potentially effective and sustainable clinical strategy for managing ADDE. This approach synchronizes treatment with the nervous system's intrinsic recovery cycles, addressing limitations of continuous stimulation paradigms.

**Trial registration:**

Chinese Clinical Trial Registry (ChiCTR2500100816). Registered on 15 April 2025 (retrospectively registered), https://www.chictr.org.cn/bin/project/edit?pid=265888.

**Supplementary Information:**

The online version contains supplementary material available at 10.1186/s40662-025-00466-w.

## Background

Dry eye disease (DED) is a highly prevalent chronic ocular surface disorder characterized by tear film instability and ocular surface inflammation [1, 2]. Its global prevalence ranges from 5% to 50%, with higher rates in specific populations, particularly women [3, 4]. In recent years, multiple factors have contributed to the increasingly reported incidence of dry eye, including increased screen time and associated blink pattern changes [5], certain medications, eye surgeries, poor environmental conditions, and contact lens use [6]. Aqueous-deficient dry eye (ADDE), a common subtype, primarily involves reduced tear secretion from the lacrimal gland [7]. Current mainstay treatments like artificial tears and anti-inflammatory drops offer symptomatic relief but face limitations including short duration of action, low bioavailability, and potential toxicity with long-term use [8–11]. Artificial tears also lack bioactive components essential for maintaining ocular surface homeostasis [12]. This unmet clinical need has spurred the development of neurostimulation therapies aimed at activating natural tear production.

Tear secretion is regulated by the lacrimal functional unit (LFU), an integrated system comprising the ocular surface (cornea, conjunctiva, meibomian glands, goblet cells), the lacrimal glands, and their interconnecting neural pathways [13]. Sensory stimulation of the ocular surface or nasal mucosa can trigger tear production via the trigeminal and parasympathetic nerves [14–16]. The nasal tear reflex is mediated by nasal mucosa stimulation. It contributes 34% of basal tear secretion [17]. Reduced nasal stimulation can decrease tear production [18]. Evidence confirms that nasal stimulation significantly enhances tear secretion [19] while concurrently promoting the secretion of lipids [20] and mucins in tear fluid [21, 22], underscoring the importance of the nasolacrimal reflex in tear film stability and the regulation of basal and reflex tear secretion.

Neuromodulatory therapies targeting the nasolacrimal reflex, such as intranasal neurostimulation therapy, have shown promise in increasing tear secretion. However, they can cause discomfort such as nosebleeds and nasal pain [23–26]. Extranasal neurostimulation presents a non-invasive alternative by stimulating the extranasal branch of the anterior ethmoidal nerve to activate the tear reflex [27]. Prior clinical investigations of extranasal neurostimulation have established its acute therapeutic effects. An initial multicenter, open-label study revealed that extranasal neurostimulation therapy can efficaciously augment basal tear secretion, with the mean Schirmer index reaching 9.4 (95% CI: 7.4 to 11.3) mm/5 min on Day 30 and the baseline ocular surface disease index (OSDI) improving by an average of 14.4 (95% CI: 11.1 to 17.7) [28]^.^ However, three critical questions remain unresolved: (1) whether neural adaptation occurs during repeated stimulation, (2) whether reduced neural sensitivity is reversible, and (3) how to design intermittent protocols to sustain long-term benefits. This knowledge gap significantly limits clinical translation, as optimal treatment cycles cannot be established without understanding these dynamic neural responses.

Our study bridges critical translational gaps by characterizing the dynamic changes in neural sensitivity, defining the recovery duration required for neural functional restoration, and establishing the first evidence based cyclic treatment protocol for extranasal neurostimulation in ADDE, and thus provides a scientific foundation for sustainable neurostimulation therapy.

## Methods

### Trial design

This prospective, open-label, randomized, parallel-group study (Chinese Clinical Trial Registry ID: ChiCTR2500100816.) was conducted at the Eye Center of Zhejiang University from October 2024 to January 2025. This study specifically aimed to (1) investigate whether repeated twice-daily stimulation (30 s per side) of the anterior ethmoidal nerve induces progressive neural sensitivity changes, (2) determine the short-term reversibility of these changes following stimulation cessation, and (3) characterize these neural dynamics and recovery patterns to establish an evidence-based treatment protocol for the clinical application of extranasal neurostimulation.

This study employed a binocular enrollment design in which both eyes of each participant were included in the analysis. Fifty eligible subjects were randomly assigned at a 1:1 ratio to either the 2-week or 4-week treatment group via a computer-generated randomization sequence (block size of 4), with allocation concealment maintained through sequentially numbered, opaque sealed envelopes. Both groups were followed for 6 weeks after stimulation cessation. Due to the difference in treatment durations, participants could not be blinded to their own treatment regimen but remained unaware of the overall trial design, including the existence of the alternative treatment duration group and study hypotheses. This approach aimed to prevent comparative bias that might arise from patients' awareness of different treatment protocols. To ensure objectivity, all efficacy assessments were performed by independent, masked evaluators following standardized protocols, with data anonymized prior to analysis. Objective indicators were prioritized as primary endpoints, while subjective questionnaires were collected by blinded personnel in a neutral setting to minimize reporting bias.

The study was approved by the Institutional Review Board of the Second Affiliated Hospital of Zhejiang University (Approval No. 2024 LXD No. 1249) and adhered to the tenets of the Declaration of Helsinki. Written informed consent was obtained from each patient before enrollment.

### Participants

The inclusion criteria for the participants were as follows: (1) age 18 years or older, with no sex restriction; (2) Schirmer I test (with topical anesthesia) result of 2–5 mm/5 min in both eyes; (3) OSDI score of ≥ 13 at the baseline visit along with one of the following signs: fluorescent tear film break-up time (FBUT) of ≤ 5 s or corneal fluorescence staining (CFS) score of ≥ 1; and (4) voluntary participation and signed consent. The exclusion criteria were as follows: (1) Sjögren's syndrome or other rheumatologic conditions; (2) recent ocular surgery (within 6 months); (3) any acute infectious or noninfectious ocular condition of the anterior or posterior segments in either eye; (4) skin infections, eczema, or other local lesions at the treatment site; (5) history of facial nerve palsy or neuromuscular disorders; and (6) concurrent enrollment in other neurostimulation or dry eye therapy trials within the 30-day prescreening period. The subjects were permitted to continue their baseline medication regimens throughout the study. All details regarding ophthalmic drops, including formulation, administration frequency, and timing, were systematically documented in case report forms.

### Investigational device and application

The iTEAR (iTEAR ARVN701-V2.0, Arctic Vision, China) is a specialized physiotherapy device designed for the treatment of dry eye. It activates the tear reflex by physically vibrating the extranasal branch of the anterior ethmoidal nerve, thereby promoting the secretion of natural tear fluid. It incorporates an oscillating tip with a frequency of approximately 250–290 Hz and an amplitude of approximately 0.4–0.8 mm. These specific parameters were selected based on the optimized and FDA-cleared predecessor device (iTEAR100) and prior systematic research by the manufacturer, which determined this range to be optimal for effectively activating neural endings while minimizing potential skin irritation or discomfort [28]. All devices were calibrated by the manufacturer (Arctic Vision) prior to shipment to ensure consistent output parameters (frequency and amplitude) in accordance with product specifications. A complete treatment session involves bilateral nasal application, with a recommended duration of 30 s per side. The device is positioned on the nasal skin at the junction of the nasal bone and cartilage, where the external nasal nerve exits the lateral nose. During the application, the subject can fine-tune the position of the vibrating head to find the optimal stimulation point to feel natural tear production more effectively.

Before starting the treatment, the researcher provided detailed instructions to the subject. The subjects were subsequently provided with a home-use device and clear instructions to use it strictly twice a day to ensure the continuity and stability of the treatment. To ensure treatment compliance, study coordinators conducted twice-weekly structured telephone follow-ups to review usage patterns and provide guidance. Additionally, all subjects were required to maintain daily treatment diaries, which were verified during each clinic visit.

### Research visits and assessments

This study employed a parallel-group design with distinct evaluation timelines for the 2-week and 4-week neurostimulation groups. Both groups underwent comprehensive assessments at six time points: baseline (Day 0), during the stimulation period (Visit 1: Day 7 for both groups), at stimulation completion (Visit 2: Day 14 for the 2-week group/Day 28 for the 4-week group), and during post-cessation follow-up (Visit 3: Day 28 for the 2-week group/Day 42 for the 4-week group; Visit 4: Day 42 for the 2-week group/Day 56 for the 4-week group; Visit 5: Day 56 for the 2-week group/Day 70 for the 4-week group).

This study established two coprimary endpoints: (1) neural sensitivity change (intra-visit Δ Schirmer): calculated as the difference between post- and pre-stimulation Schirmer I test values at the same visit time point, reflecting dynamic lacrimal gland reflex activity; (2) short-term neural recovery: defined by concurrent fulfillment of two criteria: (i) significant post-stimulation Schirmer improvement at Visit 5 (Δ post–pre, *P* < 0.05), and (ii) post-stimulation values at Visit 5 statistically equivalent to baseline (*P* ≥ 0.05). The primary neural sensitivity assessments (pre- and post-stimulation Schirmer I tests) were conducted specifically at baseline, Visits 1, 2 and 5 to evaluate acute treatment responses. At Visits 3 and 4 (stimulation-free observational periods), only pre-stimulation Schirmer I test was used to monitor natural disease progression without stimulation interference.

The secondary goals of this study were to compare improvements in dry eye symptoms and signs across different treatment durations, including pre-stimulation Schirmer I test, FBUT, tear meniscus height (TMH), non-invasive tear film break-up time (NIBUT), bulbar redness, CFS, meibomian gland expression score (MGES), meibomian gland secretion quality score (MGSQS), OSDI, and standard patient evaluation of eye dryness (SPEED), which were evaluated at all visits to provide comprehensive ocular surface assessment. The minimal clinically important difference (MCID), representing the smallest change in a score that patients perceive as beneficial, was also assessed for subjective endpoints. Based on established literature, the MCID thresholds were defined as a reduction of ≥ 10 points for the OSDI and ≥ 4 points for the SPEED questionnaire [29]. Safety and treatment satisfaction were specifically assessed at Visit 2.

### Data analysis

The sample size was determined a priori using G*Power software (version 3.1.9.7). The calculation was primarily based on the large effect size (Cohen's d = 1) reported in a pivotal study of the predecessor neurostimulation device (iTEAR100). To ensure a robust and conservative estimate, a large effect size (Cohen's d = 0.85) was selected for this study. A minimum of 44 participants (22 per group) were required to achieve 90% power at α = 0.05 (two-tailed t-test). To account for a potential 15% attrition rate, 50 subjects (25 per group) were enrolled.

Statistical analyses were performed via SPSS software (version 26.0; IBM Corp, Armonk, NY, USA) and GraphPad Prism (version 10.0; GraphPad Software, San Diego, CA, USA). Normally distributed variables are presented as the mean ± standard deviation, whereas categorical variables are presented as values or percentages. The independent sample t-test was used for intergroup comparisons. Paired sample t-test was applied for comparisons between pre- and post-stimulation measurements at each time point. Within-group changes in continuous variables (baseline vs. follow-up) were analyzed via Bonferroni-corrected repeated-measures ANOVA. The Mauchly’s test of sphericity was performed, and when violated, the Greenhouse–Geisser correction was applied. Effect sizes for ANOVA are reported as partial eta-squared (η^2^p). For intergroup comparisons using t-tests, effect sizes are reported as Cohen's d. The proportion of patients achieving the MCID at each time point was compared within groups using Cochran’s Q test and between groups using Chi-squared tests. All the statistical tests were two-sided, and *P* values less than 0.05 were considered statistically significant.

## Results

### Subject disposition and baseline characteristics

**Fig. 1:**
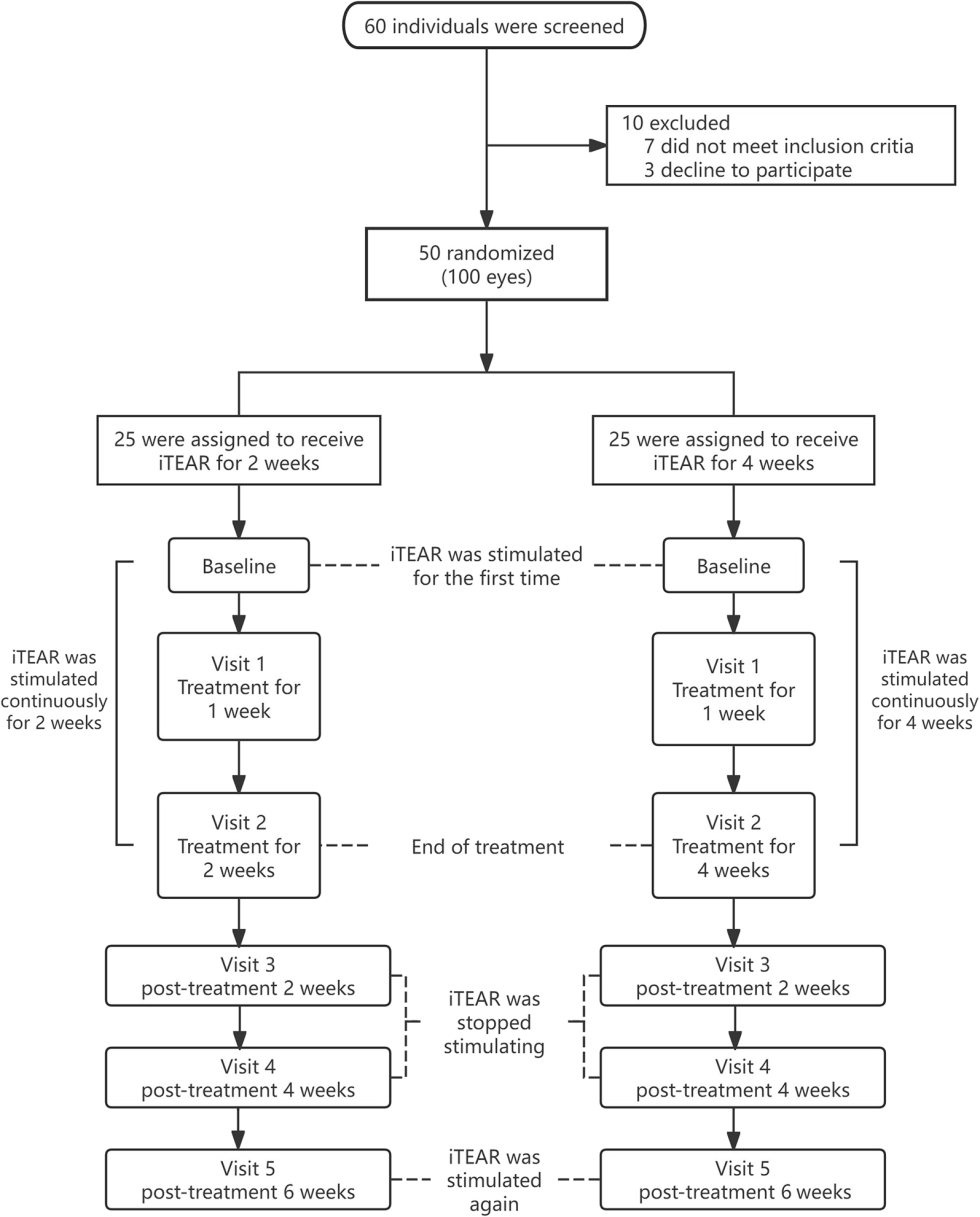
Trial design and participant flow. Schematic diagram of the study protocol. Eligible subjects with aqueous-deficient dry eye were randomized to receive 2-week or 4-week bilateral extranasal neurostimulation (twice daily). Both groups were followed for six weeks after treatment cessation. Comprehensive ocular surface assessments were performed at the indicated time points (baseline, Visits 1–5). Schirmer I tests (with anesthesia) were performed both pre- and post-stimulation at baseline, Visits 1, 2, and 5, and only pre-stimulation at Visits 3 and 4 to monitor natural disease progression

A total of 60 prospective participants were screened, with 7 excluded and 3 withdrawn. A total of 50 volunteers, including 39 women and 11 men, with an average age of 32.02 ± 7.16 years, completed the follow-up. Twenty-five people in each of the 2-week treatment groups and the 4-week treatment group completed the follow-up. The treatment and follow-up protocols for the two groups are shown in Fig. 1. Subject demographics and clinical characteristics were comparable between the 2-week and 4-week treatment groups (Table 1). All subjects had non-autoimmune ADDE.

**Table 1:** Comparison of baseline characteristics between groups

Characteristics	2-week group (n = 25)	4-week group (n = 25)	Statistical test	*P* value
Demographics				
Eyes, n	50	50	–	–
Age, years	33.04 ± 7.54	31.00 ± 6.57	t = 1.008	0.318
Sex, male:female	4:21	7:18	*χ*^*2*^ = 1.049	0.306
Dry eye endpoints				
OSDI score	31.03 ± 17.25	40.07 ± 18.09	t = − 1.809	0.077
Schirmer (mm)	3.40 ± 1.23	3.28 ± 1.11	t = 0.513	0.609
TMH (mm)	0.19 ± 0.03	0.19 ± 0.02	t = 0.556	0.580
FBUT (s)	2.86 ± 1.01	3.09 ± 1.13	t = − 1.092	0.278
SPEED score	11.32 ± 6.02	12.68 ± 5.14	t = − 0.859	0.394
NIBUT (s)	7.28 ± 4.32	7.59 ± 5.03	t = − 0.329	0.743
Bulbar redness	1.09 ± 0.35	1.10 ± 0.29	t = 0.551	0.925
CFS score	0.40 ± 1.14	0.34 ± 0.87	t = 0.295	0.768
MGES	0.50 ± 0.97	0.34 ± 0.72	t = 0.935	0.352
MGSQS	1.84 ± 3.33	1.76 ± 2.84	t = 0.129	0.898

*OSDI* = ocular surface disease index; *TMH* = tear meniscus height; *FBUT* = fluorescein tear breakup time; *SPEED* = standard patient evaluation of eye dryness; *NIBUT* = noninvasive breakup time; *CFS* = corneal fluorescein staining; *MGES* = meibomian gland expression score; *MGSQS* = meibomian gland secretion quality score

Continuous variables are presented as the means ± standard deviations and were compared via independent sample *t-test;* categorical variables (sex) were analyzed via the *χ*^*2*^ test

### Primary endpoint: neural sensitivity changes and recovery

Initial Stimulation Response: Both groups showed a robust initial response to neurostimulation at baseline. The Schirmer score increased significantly from 3.40 ± 1.23 to 14.88 ± 8.69 mm/5 min (Δ = 11.48 ± 8.23 mm/5 min, 95% CI: 9.14 to 13.82 mm/5 min, *P* < 0.001, d = 1.39) in the 2-week group and from 3.28 ± 1.11 to 12.42 ± 6.67 mm/5 min (Δ = 9.14 ± 6.68 mm/5 min, 95% CI: 7.24 to 11.04 mm/5 min, *P* < 0.001, d = 1.37) in the 4-week group. The between-group difference in the initial response was not significant (*P* = 0.122, Cohen’s d = 0.31).

**Fig. 2:**
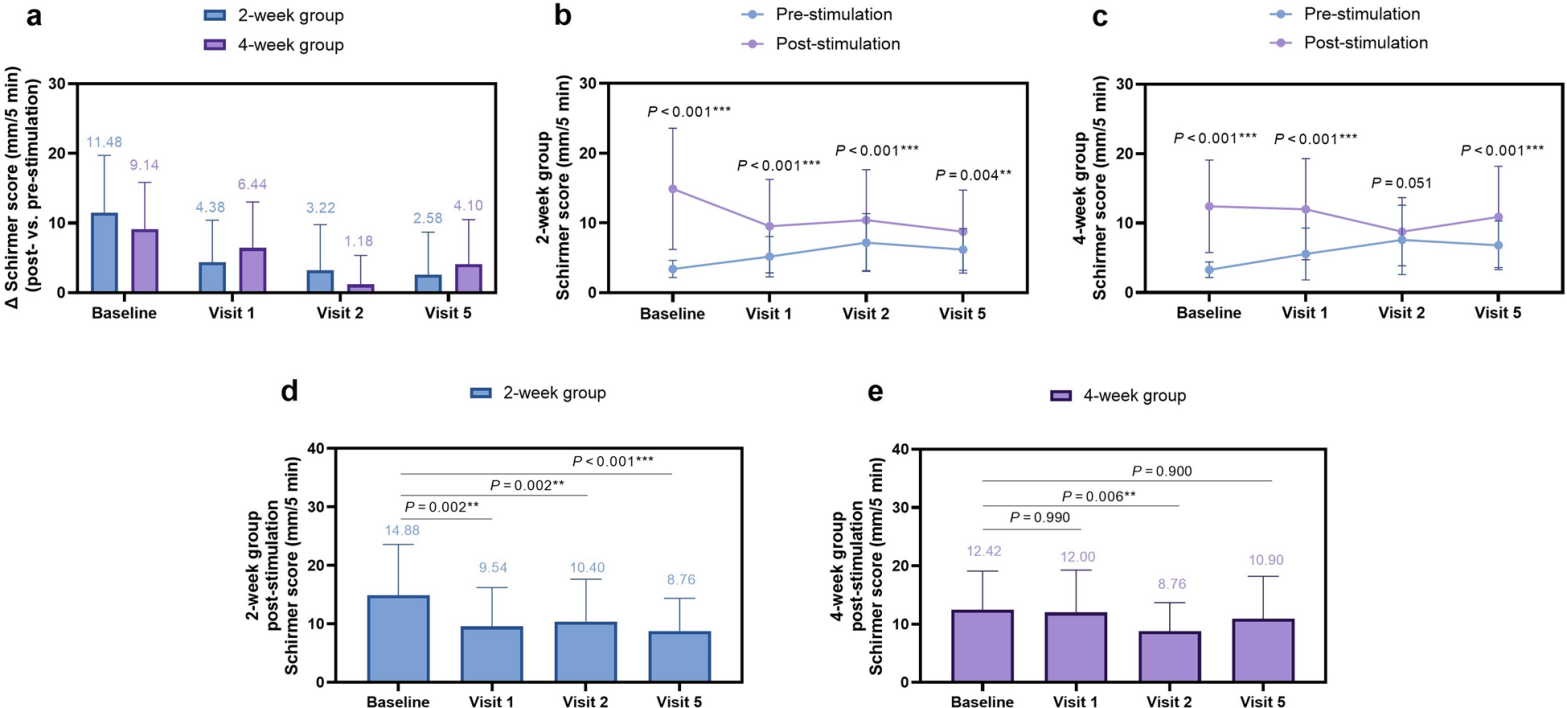
Dynamic changes in neural sensitivity and acute tear secretion response.** a** Mean change in Schirmer scores (post- vs. pre-stimulation) over time, illustrating the attenuation of the acute reflex response in the 4-week group;** b** Pre- and post-stimulation Schirmer scores across visits in the 2-week group, showing preserved responsiveness during treatment but persistent hyposensitivity at Visit 5; **c** Pre- and post-stimulation Schirmer scores across visits in the 4-week group, demonstrating neural adaptation at Visit 2 and complete recovery of responsiveness at Visit 5; **d** Post-stimulation Schirmer scores at key visits in the 2-week group, showing values at Visit 5 significantly below the initial response; **e** Post-stimulation Schirmer scores at key visits in the 4-week group, showing values at Visit 5 comparable to the initial response. Data are presented as mean ± SD. **P < 0.01, ***P < 0.001 for within-group pre- vs. post-stimulation comparison at the same visit (**b**,** c**) or for comparison vs. baseline post-stimulation value (**d**, **e**)

Sustained Stimulation Phase: Divergent patterns emerged with continued stimulation (Table 2, Fig. 2a). The 2-week group maintained significant responsiveness at treatment completion (Visit 2, Day 14) with Schirmer scores increased from 7.18 ± 4.14 to 10.40 ± 7.25 mm/5 min post-stimulation (Δ = 3.22 ± 6.54 mm/5 min, 95% CI: 1.33 to 5.11 mm/5 min, *P* < 0.001, Cohen’s d = 0.49; Fig. 2b). Conversely, the 4-week group exhibited a strong trend toward neural adaptation at treatment completion (Visit 2, Day 28), where the mean increase in tear secretion was markedly attenuated and no longer reached conventional statistical significance (7.58 ± 4.98 mm/5 min pre-stimulation vs. 8.76 ± 4.92 mm/5 min post-stimulation, Δ = 1.18 ± 4.17 mm/5 min, 95% CI: − 0.01 to 2.37 mm/5 min, *P* = 0.051, Cohen's d = 0.28*; *Fig. 2c).

**Table 2:** Dynamic changes in tear secretion in response to extranasal neurostimulation: a comparison between 2-week and 4-week treatment protocols

Visit	Group	Pre-stimulation (mm/5 min)	Post-stimulation (mm/5 min)	Change (Δ) (mm/5 min)	95% CI of Δ	Within-group *P* (Cohen's d)	Δ Between-group difference (95% CI)	Δ between-group *P* (Cohen's d)
Baseline (Day 0)	2-week	3.40 ± 1.23	14.88 ± 8.69	11.48 ± 8.23	9.14–13.82	< 0.001^***^ (d = 1.39)	− 0.63–5.31	0.122 (d = 0.31)
	4-week	3.28 ± 1.11	12.42 ± 6.67	9.14 ± 6.68	7.24–11.04	< 0.001^***^ (d = 1.37)		
Visit 1 (Day 7)	2-week	5.16 ± 2.89^††^	9.54 ± 6.69^##^	4.38 ± 6.03	2.67–6.09	< 0.001^***^ (d = 0.73)	− 4.56–0.44	0.105 (d = 0.33)
	4-week	5.56 ± 3.74^†††^	12.00 ± 7.28	6.44 ± 6.57	4.57–8.30	< 0.001^***^ (d = 0.98)		
Visit 2 (Day 14/28)	2-week	7.18 ± 4.14^†††^	10.40 ± 7.25^##^	3.22 ± 6.54	1.33–5.11	< 0.001^***^ (d = 0.49)	− 0.14–4.22	0.066 (d = 0.37)
	4-week	7.58 ± 4.98^†††^	8.76 ± 4.92^##^	1.18 ± 4.17	− 0.01–2.37	0.051 (d = 0.28)		
Visit 3 (Day 28/42)	2-week	6.68 ± 4.18^†††^	–	–	–	–	–	–
	4-week	6.98 ± 4.69^†††^	–	–	–	–		
Visit 4 (Day 42/56)	2-week	6.30 ± 3.52^†††^	–	–	–	–	–	–
	4-week	6.44 ± 3.54^†††^	–	–	–	–		
Visit 5 (Day 56/70)	2-Week	6.18 ± 2.99^†††^	8.76 ± 5.95^###^	2.58 ± 6.10	0.84–4.31	0.004^**^ (d = 0.42)	− 4.00–0.96	0.226 (d = 0.24)
	4-Week	6.80 ± 3.52^†††^	10.90 ± 7.29	4.10 ± 6.38	2.29–5.91	< 0.001^***^ (d = 0.64)		

Data are presented as Mean ± SD

P** < 0.01, P*** < 0.001 for within-group pre- versus post-stimulation comparison

*P*^##^ < 0.01, *P*^###^ < 0.001 for post-stimulation value versus baseline post-stimulation value

*P*^††^ < 0.01, *P*^†††^ < 0.001 for pre-stimulation value versus baseline post-stimulation value

Effect sizes for within-group pre-post changes are reported as Cohen's d

Visits 3 and 4 were observational periods; only pre-stimulation assessments were performed

*CI* = confidence interval

Stimulation Cessation Phase: At the 6-week post-cessation (Visit 5), the 4-week group demonstrated complete neural recovery. The post-stimulation Schirmer values (10.90 ± 7.29 mm/5 min) were comparable to the initial post-stimulation response at baseline (12.42 ± 6.67 mm/5 min, 95% CI: − 4.38 to 1.34 mm/5 min, *P* = 0.90, η^2^p = 0.092; Table 2, Fig. 2e) while still eliciting a significant acute response compared to pre-stimulation values at Visit 5 (Δ = 4.10 ± 6.38 mm/5 min, 95% CI: 2.29 to 5.91 mm/5 min, *P* < 0.001*,* Cohen’s d = 0.64; Fig. 2c). In contrast, the 2-week group showed persistent hyposensitivity at Visit 5. Although a significant acute response was still present (post-stimulation 8.76 ± 5.95 mm/5 min vs. pre-stimulation 6.18 ± 2.99 mm/5 min; Δ = 2.58 ± 6.10 mm/5 min, 95% CI: 0.84 to 4.31 mm/5 min, *P* = 0.004, Cohen’s d = 0.42; Fig. 2b), the absolute post-stimulation values remained significantly lower than the baseline post-stimulation level (14.88 ± 8.69 mm/5 min, 95% CI: − 9.70 to − 2.54 mm/5 min, *P* < 0.001, η^2^p = 0.24; Fig. 2d).

### Secondary outcomes and safety analysis

**Fig. 3:**
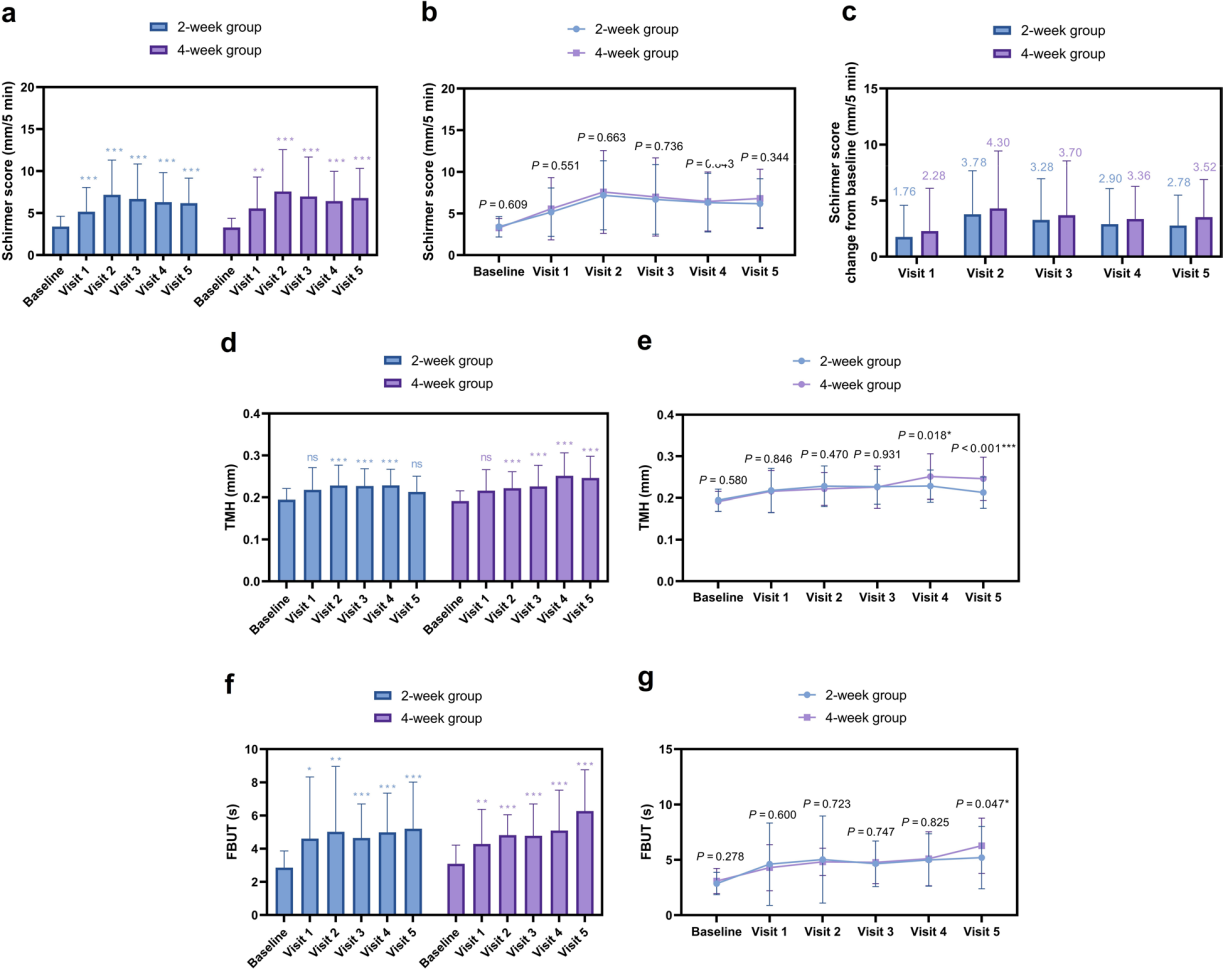
Comparative analysis of tear secretion volume and tear film stability.** a** Mean pre-stimulation Schirmer scores over time, reflecting basal tear secretion; **b** Change in pre-stimulation Schirmer scores from baseline;** c** Between-group differences in pre-stimulation Schirmer scores; **d** Mean tear meniscus height (TMH) over time; **e** Between-group differences in TMH; **f** Mean fluorescein tear film break-up time (FBUT) over time; **g** Between-group differences in FBUT. Data are presented as mean ± SD. *P < 0.05, **P < 0.01, ***P < 0.001 for within-group comparison vs. baseline (**a**, **d**, **f**) or between-group comparison at the same visit (**b**, **e**, **g**). ns, not significant

Tear Secretion and Stability: Basal tear secretion (pre-stimulation Schirmer) improved significantly from baseline in both groups (2-week group: *P* < 0.001, η^2^p = 0.237, 4-week group: *P* < 0.001, η^2^p = 0.186; Fig. 3a), with a non-significant trend favoring the 4-week group (*P* = 0.226, Cohen’s d = 0.19; Fig. 3b, c). TMH showed sustained improvement throughout the 6-week follow-up in the 4-week group but progressively declined in the 2-week group, returning to baseline levels by Visit 5 (Δ = 0.02 ± 0.04 mm, *P* = 0.11, η^2^p = 0.112; Fig. 3d). Between-group differences reached significance at 4-week and 6-week post-cessation (4-week: *P* = 0.018, Cohen’s d = 0.48; 6-week: *P* < 0.001, Cohen’s d = 0.74; Fig. 3e). FBUT improved significantly in both groups (2-week group: *P* < 0.001, η^2^p = 0.094, 4-week group: *P* < 0.001, η^2^p = 0.235; Fig. 3f), with the 4-week group maintaining superior FBUT values compared to the 2-week group at the 6-week post-cessation visit (6.27 ± 2.49 s vs. 5.20 ± 2.81 s, *P* = 0.047, Cohen’s d = 0.40; Fig. 3g).

**Fig. 4:**
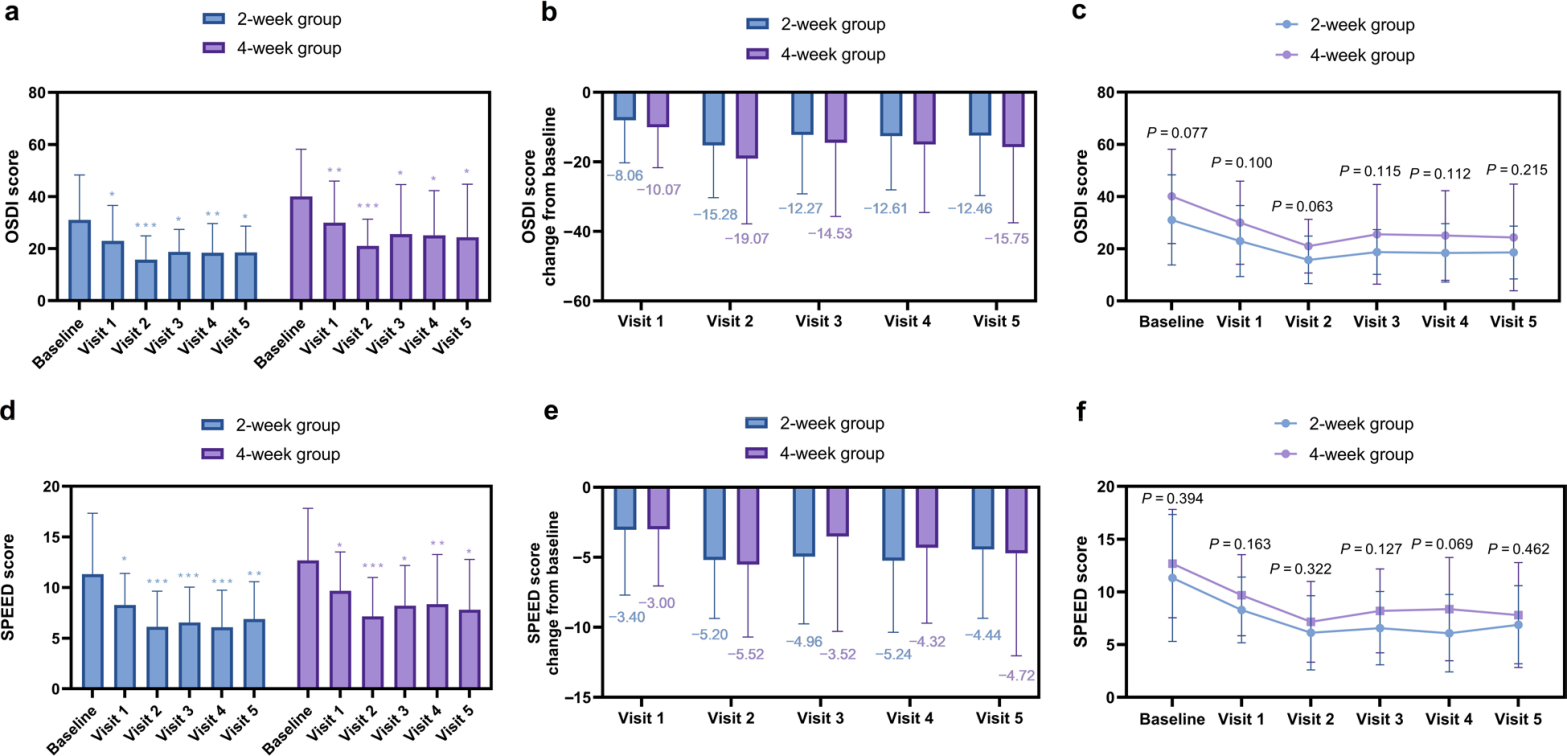
Comparative analysis of subjective symptoms.** a** Mean ocular surface disease index (OSDI) scores over time; **b** Change in OSDI score from baseline; **c** Between-group differences in OSDI scores; **d** Mean standard patient evaluation of eye dryness (SPEED) scores over time; **e** Change in SPEED score from baseline; **f** Between-group differences in SPEED scores. Data are presented as mean ± SD. *P < 0.05, **P < 0.01, ***P < 0.001 for within-group comparison vs. baseline

Subjective Symptoms: Both OSDI and SPEED scores improved significantly from baseline in both groups (OSDI: 2-week group: *P* = 0.02, η^2^p = 0.313, 4-week group: *P* = 0.03, η^2^p = 0.262, Fig. 4a; SPEED: 2-week group: *P* = 0.002, η^2^p = 0.366, 4-week group: *P* = 0.011, η^2^p = 0.239, Fig. 4d). Although the between-group differences in mean change scores did not reach statistical significance (OSDI: *P* = 0.215, Cohen’s d = 0.36, Fig. 4c***;*** SPEED: *P* = 0.462, Cohen’s d = 0.22, Fig. 4f, the 4-week group consistently showed a greater magnitude of improvement in both subjective symptom scores compared to the 2-week group (Fig. 4b, d).

This trend toward enhanced benefit in the 4-week group was further supported by MCID-based responder analysis. A higher proportion of patients in the 4-week group achieved clinically meaningful improvement at the end of treatment for both OSDI (72% vs. 60%) and SPEED (64% vs. 56%). Although these differences in MCID rates did not reach statistical significance (Table 3), the consistent directional trend across both subjective endpoints reinforces the potential clinical relevance of the longer treatment duration. Longitudinal assessment revealed that the 4-week group exhibited more stable MCID responder rates over time for both OSDI and SPEED. In contrast, the 2-week group showed significant fluctuation in OSDI responder rates (*P* = 0.032 by Cochran's Q test), while its SPEED responder rates remained relatively stable (*P* = 0.525).

**Table 3:** Comparison of the proportion of patients achieving the minimal clinically important difference (MCID) at each visit

Indicator	Group	Visit 1 (Day 7)	Visit 2 (Day 14/28)	Visit 3 (Day 28/42)	Visit 4 (Day 42/56)	Visit 5 (Day 56/70)	*P* value^a^
OSDI	2-week	8 (32%)	15 (60%)	12 (48%)	15 (60%)	14 (56%)	0.032*
	4-week	14 (56%)	18 (72%)	16 (64%)	16 (64%)	14 (56%)	0.323
	*P* value^b^	0.087	0.370	0.254	0.771	1.000	
SPED							
	2-week	10 (40%)	14 (56%)	14 (56%)	12 (48%)	12 (48%)	0.525
	4-week	10 (40%)	16 (64%)	15 (60%)	14 (56%)	13 (52%)	0.199
	*P* value^b^	1.000	0.574	0.774	0.571	0.771	

Data are presented as n (%)

a: * P* value from Cochran's Q test, comparing the proportion achieving MCID across all time points within each group P* < 0.05

b: *P* value from Chi-squared test, for the difference in proportions between groups at each time point

*OSDI* = ocular surface disease index; *SPED* = symptom perception in dry eye disease

**Fig. 5:**
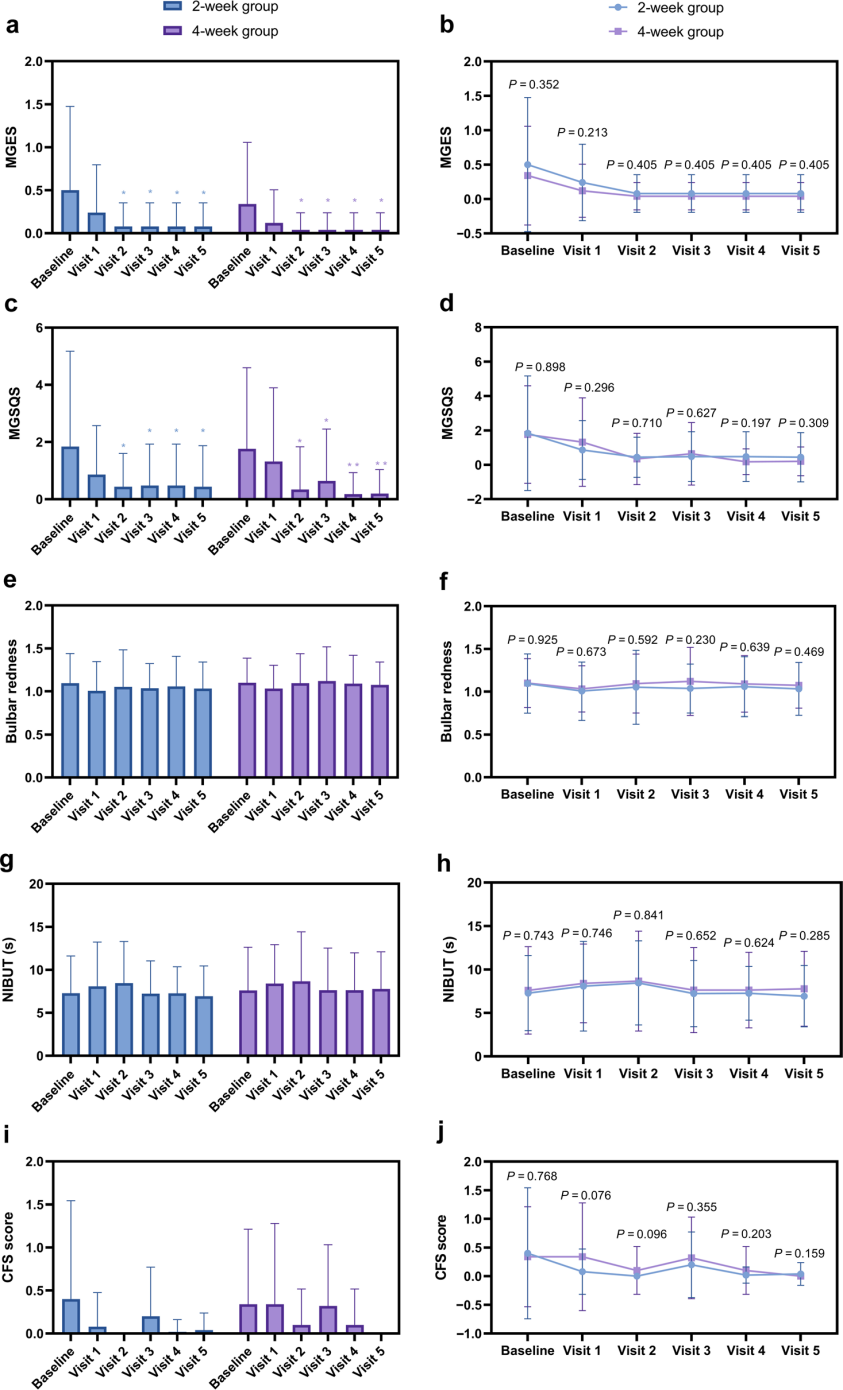
Comparative analysis of meibomian gland function and other ocular surface parameters. **a** Mean meibomian gland expression score (MGES); **b** Change in MGES from baseline. **c** Mean meibomian gland secretion quality score (MGSQS). **d** Change in MGSQS from baseline. **e** Mean bulbar redness score. **f** Change in bulbar redness from baseline. **g** Mean non-invasive tear film break-up time (NIBUT); **h** Change in NIBUT from baseline. **i** Mean corneal fluorescein staining (CFS) score; **j** Change in CFS score from baseline. Data are presented as mean ± SD. *P < 0.05, **P < 0.01 for within-group comparison vs. baseline. No statistically significant changes from baseline were observed for bulbar redness, NIBUT, or CFS within or between groups

Meibomian Gland Function and Other Parameters: MGES and MGSQS improved similarly in both groups (Fig. 5a–d). No significant changes were observed in NIBUT, bulbar redness, or CFS scores within or between groups (Fig. 5e–j).

Safety and Satisfaction: The safety profiles were similar (see Additional file 1), with no severe adverse events. Treatment assessments revealed 84% satisfaction (see Additional file 2), whereas stimulation tolerability data indicated that 50% reported no discomfort and that 40% required adaptation (see Additional file 3). Efficacy outcomes included 30% improvement, 26% no change, and 44% reduced effectiveness (see Additional file 4).

## Discussion

DED remains a significant therapeutic challenge because of its ocular surface inflammation and tear film instability [30]. Extranasal neurostimulation has emerged as a promising neuromodulatory approach that targets the nasolacrimal reflex pathway to restore tear film homeostasis [28]. However, the clinical application of this therapy has been hampered by the lack of evidence-based guidance regarding the optimal treatment duration and cycling protocols. This study elucidates the dynamic changes in neural sensitivity during extranasal neurostimulation, and based on these dynamics, we propose an optimized cyclic regimen of 4-week stimulation followed by a 6-week intermission. This protocol is designed to harness the nervous system's innate adaptive rhythms, balancing maximal therapeutic efficacy with long-term sustainability.

Our study delineates a critical time-dependent relationship between extranasal neurostimulation duration and neural adaptation within the LFU. While a 2-week stimulation period sustained neural reactivity, extending treatment to 4 weeks induced significant neural desensitization, evidenced by a markedly attenuated tear secretion response. This duration-dependent pattern is consistent with Benda’s neural adaptation theory [31] and mirrors response attenuation observed in other neuromodulatory models, such as repetitive transcranial magnetic stimulation (rTMS) [32] and visual cortical stimulation [33]. Our results extend these principles to the nasolacrimal reflex pathway, demonstrating that the LFU shares fundamental adaptive properties with other neural circuits. This identification of a 4-week stimulation period as the threshold for full adaptive response provides the first fundamental parameter for our cyclic regimen. Although the precise mechanisms remain to be fully elucidated, we hypothesize that common neural adaptive mechanisms such as synaptic neurotransmitter depletion [34] and receptor desensitization [35] may underlie this effect, though this remains speculative in the context of the anterior ethmoidal nerve. Consequently, to sustain treatment benefits while respecting adaptive processes, clinical implementation should prioritize intermittent cycles with a defined stimulation period not extending beyond this 4-week threshold.

The neural system exhibited intrinsic short-term plasticity, characterized by duration-dependent adaptation and spontaneous recovery. We observed this plasticity as a progressive reduction in neural sensitivity during the 4-week stimulation period, with complete functional recovery occurring after a 6-week intermission. This recovery pattern likely reflects the nervous system's capacity to return to baseline through post-processing biological processes, including neurotransmitter resynthesis and receptor resensitization. The comprehensive reversibility of these adaptive changes confirms that four weeks of stimulation induces physiological neural modulation rather than pathological damage, thereby substantiating the safety profile of cyclic treatment applications. The consistent and complete functional restoration observed specifically at the 6-week time point defines the optimal duration of the treatment intermission. This allows the neural pathway to fully reset its sensitivity, thereby ensuring that the subsequent stimulation cycle can elicit a robust therapeutic response identical to the initial treatment. In contrast, the 2-week protocol failed to engage this compensatory neuroplasticity, resulting in persistent hyposensitivity after cessation. For clinicians, these temporal dynamics indicate that a full 4-week course of stimulation must be paired with a 6-week recovery period. This specific combination is critical to transitioning from short-term symptom relief to a sustainable long-term management strategy, as shorter regimens fail to produce the lasting adaptive changes needed for cyclic therapy.

Comparative analysis confirmed that extranasal neurostimulation effectively enhances multiple components of tear film function. The treatment significantly improved basal tear secretion in both groups, confirming its fundamental efficacy in enhancing aqueous production. Beyond aqueous enhancement, the therapy also improved mucin function, as reflected by enhanced tear film stability (increased FBUT) and correlative improvements in ocular surface protection, and positively influenced lipid layers through improved meibomian gland function (MGES and MGSQS scores), despite not specifically enrolling patients with meibomian gland dysfunction. Supported by established mechanisms, enhanced mucin secretion can improve the stability of the tear film by providing a better lubricating and protective layer [36], whereas improved meibomian gland function can lead to more stable lipid secretion, which helps prevent tear evaporation [37]. Furthermore, the observed increases in basal tear secretion and tear film stability indicate potential ancillary anti-inflammatory mechanisms, possibly mediated through continuous dilution and clearance of inflammatory mediators via enhanced tear flow [38], as well as restoration of natural anti-inflammatory components (e.g., lactoferrin and lysozyme) in the tear film [39]. These multi-mechanistic effects position neurostimulation as a comprehensive therapeutic approach that simultaneously targets multiple deficient components in DED.

The 4-week protocol demonstrated clearly superior and more sustained benefits across both objective and subjective measures. Most notably, this extended regimen provided significantly better outcomes in FBUT and TMH, suggesting that extended stimulation may promote more lasting improvements in tear film composition and ocular surface homeostasis. Clinically, the 4-week group showed particularly compelling advantages: a higher proportion of patients achieved clinically meaningful improvement in OSDI scores (72% vs. 60%) and exhibited more stable responder rates over time, indicating not only stronger efficacy but also more consistent and durable patient benefits—crucial considerations for chronic management. Collectively, these findings provide important evidence for optimizing treatment protocols, suggesting that the 4-week stimulation regimen should be prioritized in clinical practice to achieve more comprehensive therapeutic outcomes through simultaneous enhancement of aqueous production, lipid stability, mucin function, and potential anti-inflammatory effects.

Our findings establish a paradigm shift in dry eye management by introducing an evidence-based cyclic regimen of extranasal neurostimulation, which offers distinct advantages over existing treatment paradigms. This protocol offers a novel non-pharmacologic alternative to the conventional ADDE treatment algorithm which relies on escalating steps from artificial tears and anti-inflammatories to secretagogues or procedural interventions [7]. While foundational, these options face challenges including variable efficacy, frequent dosing, side effects of chronic pharmacotherapy, and irreversibility. Our study introduces extranasal neurostimulation as a non-pharmacologic alternative that activates the nasolacrimal reflex to promote natural tear production. The cyclic regimen aligns with the goal of restoring physiological secretion while avoiding limitations of chronic topical drugs, offering a sustainable option particularly suited for patients seeking drop-free alternatives or with suboptimal response to conventional treatments. While existing neurostimulation approaches such as TrueTear [40] and Tyrvaya [41] employ continuous daily use without considering neural adaptation dynamics, a limitation that significantly restricts their long-term applicability for chronic dry eye, our work uniquely establishes the critical relationship between extranasal stimulation duration, neural adaptation dynamics, and clinical outcomes. The common continuous-use strategy, which ignores the phenomenon of neural desensitization, likely explains the diminished long-term efficacy observed in some studies [24]. In contrast, our intermittent strategy is precisely timed to preempt neural desensitization by concluding stimulation at the 4-week mark, and to leverage the system's innate recovery cycle by allowing a 6-week intermission. This approach preserves long-term responsiveness and enhances therapeutic sustainability by working in concert with, rather than against, the body's physiological patterns. Methodologically, our study design offers key strengths over prior neurostimulation trials. Unlike previous trials that employed responder-enrichment designs (requiring ≥ 10 mm Schirmer improvement for enrollment) [28], our trial enrolled an unselected real-world population with moderate aqueous deficiency (baseline Schirmer 2–5 mm). This inclusive approach, coupled with our 100% retention rate, minimizes selection and survivor biases that have potentially inflated efficacy estimates in earlier studies. By demonstrating that a defined period of stimulation induces reversible neural plasticity and yields sustained benefits, our work provides the first mechanistic framework for cyclic application of neurostimulation, offering a promising new strategy for long-term dry eye management.

However, several limitations of this study should be considered when interpreting these findings. First, as a single-center investigation, our findings may be influenced by the specific healthcare environment and patient population, and caution should be exercised when extrapolating the conclusions to dry eye patients in different regions or under different clinical settings. Second, the relatively small sample size may limit the statistical power to detect smaller between-group differences and preclude meaningful subgroup analyses, making it difficult to clarify the neural adaptation patterns and therapeutic characteristics across different populations. Third, this study evaluated neural adaptation and recovery patterns only within a single “stimulation-intermission” cycle. The proposed cyclic regimen is therefore a proof-of-concept based on single-cycle dynamics; the long-term efficacy, sustainability of neural adaptation, and potential cumulative effects over repeated cycles remain unknown. Fourth, the inference of neural adaptation was based solely on indirect clinical measures (Schirmer test) rather than direct neurophysiological assessments such as electroneurography, corneal sensitivity measurements, in vivo confocal microscopy, or tear neurotransmitter analysis. Therefore, the underlying molecular and cellular mechanisms remain unclear. Additionally, corneal sensitivity—a key clinical indicator of trigeminal nerve function—was not assessed. Fifth, the stimulation parameters used were fixed; future dose-ranging studies are needed to examine whether different parameters (e.g., frequency, amplitude) could enhance efficacy or modulate adaptation kinetics.

Future studies should address the current limitations through several targeted approaches: large-scale, multicenter trials to validate the effectiveness and safety of the “4-week stimulation + 6-week intermission” regimen in diverse populations and to investigate predictors of interindividual variability; long-term studies spanning 12–24 months to evaluate the durability of effects and neural adaptation stability over multiple cycles; direct neurophysiological and molecular assessments (e.g., electroneurography, corneal confocal microscopy, tear neurotransmitter analysis) to elucidate underlying mechanisms; and inclusion of corneal esthesiometry to comprehensively evaluate trigeminal nerve function alongside tear secretion changes.

## Conclusion

On the basis of the neural adaptation and recovery patterns observed in this study, we demonstrated that a cyclic regimen consisting of 4 weeks of stimulation followed by 6 weeks of intermission provides a mechanistic rationale and a preliminary protocol reference for the cyclic treatment of DED. This defined cycle is not arbitrary but is directly calibrated to the intrinsic recovery kinetics of the nasolacrimal reflex pathway, effectively addresses the limitations associated with continuous stimulation paradigms. However, the long-term efficacy and safety of its multi-cycle application require further validation through long-term studies.

## Supplementary Information

Additional files

## Data Availability

The datasets generated and analyzed during this study are available from the corresponding author upon reasonable request.

## Abbreviations

DED: Dry eye disease
ADDE: Aqueous-deficient dry eye
LFU: Lacrimal functional unit
OSDI: Ocular surface disease index
SPEED: Standard patient evaluation of eye dryness
MCID: Minimal clinically important difference
TMH: Tear meniscus height
FBUT: Fluorescein tear film break-up time
NIBUT: Non-invasive tear film break-up time
MGES: Meibomian gland expression score
MGSQS: Meibomian gland secretion quality score
CFS: Corneal fluorescein staining
rTMS: Repetitive transcranial magnetic stimulation
