# Supplementary material for: Cyclic extranasal neurostimulation for dry eye disease: a 4-week stimulation and 6-week intermission regimen based on neural adaptation dynamics
Source: Eye Vis (Lond). 2025 Dec 2;12:49. doi: 10.1186/s40662-025-00466-w (PMC12670825; doi:10.1186/s40662-025-00466-w)
Supplement: Supplementary file 1 — Additional files [file 40662_2025_466_MOESM1_ESM.docx]

**Additional files**

**Additional file 1.** Treatment-related symptoms and recovery times.

| **Category** | 1. **week group (n=25)**   **n (%)** | **4-week group (n=25)**  **n (%)** | **Total**  **(n=50)**  **n (%)** |
| --- | --- | --- | --- |
| Overall Discomfort |  |  |  |
| No discomfort | 10 (40%) | 5 (20%) | 15 (30%) |
| Nasal itching |  |  |  |
| Very mild | 3 (12%) | 8 (32%) | 11 (22%) |
| Mild but tolerable | 10 (40%) | 12 (48%) | 22 (44%) |
| Severe | 0 (0%) | 0 (0%) | 0 (0%) |
| Sneezing |  |  |  |
| Very mild | 0 (0%) | 3 (12%) | 3 (6%) |
| Mild but tolerable | 5 (20%) | 7 (28%) | 12 (24%) |
| Severe | 0 (0%) | 0 (0%) | 0 (0%) |
| Recovery time |  |  |  |
| 1–3 days | 5 (20%) | 7 (28%) | 12 (24%) |
| 3–7 days | 0 (0%) | 2 (8%) | 2 (4%) |
| >7 days | 0 (0%) | 1 (4%) | 1 (2%) |

**Additional file 2.** Patient satisfaction outcomes.

| **Group** | **Very satisfied**  **n (%)** | **Somewhat satisfied**  **n (%)** | **Neutral**  **n (%)** | **Dissatisfied**  **n (%)** | **Very dissatisfied**  **n (%)** |
| --- | --- | --- | --- | --- | --- |
| 2-week (n=25) | 14 (56%) | 9 (36%) | 2 (8%) | 0 (0%) | 0 (0%) |
| 4-week (n=25) | 7 (28%) | 14 (56%) | 4 (16%) | 0 (0%) | 0 (0%) |
| Total  (n=50) | 21 (42%) | 23 (46%) | 6 (12%) | 0 (0%) | 0 (0%) |

**Additional file 3.** Device tolerability and usability metrics.

| **Comfort level during stimulation** | | | | | | |
| --- | --- | --- | --- | --- | --- | --- |
| Group | Very comfortable  n (%) | No discomfort (acceptable)  n (%) | | Some discomfort (acceptable after adaptation)  n (%) | | Very uncomfortable (unacceptable)  n (%) |
| 2-week (n=25) | 5 (20%) | 10 (40%) | | 10 (40%) | | 0 (0%) |
| 4-week (n=25) | 0 (0%) | 15 (60%) | | 10 (40%) | | 0 (0%) |
| Total (n=50) | 5 (10%) | 25 (50%) | | 20 (40%) | | 0 (0%) |
| **Stimulation duration patterns** | | | | | | |
| Group | ≤10 s/side  n (%) | 10–20 s/side  n (%) | | 20–30 s/side  n (%) | | 30 s No response  n (%) |
| 2-week (n=25) | 0 (0%) | 4 (16%) | | 21 (84%) | | 0 (0%) |
| 4-week (n=25) | 0 (0%) | 4 (16%) | | 21 (84%) | | 0 (0%) |
| Total (n=50) | 0 (0%) | 8 (16%) | | 42 (84%) | | 0 (0%) |
| **Moderate difficulty** | | | | | | |
| Group | Very easy to locate  n (%) | | Can be found after exploration  n (%) | | Very difficult to locate  n (%) | |
| 2-week (n=25) | 11 (44%) | | 14 (56%) | | 0 (0%) | |
| 4-week (n=25) | 7 (28%) | | 17 (68%) | | 1 (4%) | |
| Total (n=50) | 18 (36%) | | 31 (62%) | | 1 (2%) | |

**Additional file 4.** Longitudinal efficacy assessment.

| **Perceived change in treatment effect over time** | | | | | | |
| --- | --- | --- | --- | --- | --- | --- |
| Group | Effect improves over time  n (%) | | Little change  n (%) | | Less effective than initial use  n (%) | |
| 2-week (n=25) | 10 (40%) | | 6 (24%) | | 9 (36%) | |
| 4-week (n=25) | 5 (20%) | | 7 (28%) | | 13 (52%) | |
| Total (n=50) | 15 (30%) | | 13 (26%) | | 22 (44%) | |
| **Adjustment of other dry eye treatment frequency after iTEAR use** | | | | | | |
| Group | | No other eye drops used  n (%) | | Increased eye drop use  n (%) | | Decreased eye drop use  n (%) |
| 2-week (n=25) | | 24 (96%) | | 1 (4%) | | 0 (0%) |
| 4-week (n=25) | | 18 (72%) | | 2 (8%) | | 5 (20%) |
| Total (n=50) | | 42 (84%) | | 3 (6%) | | 5 (10%) |
